# Identification of *qBK2.1*, a novel QTL controlling rice resistance against *Fusarium fujikuroi*

**DOI:** 10.1186/s40529-023-00375-y

**Published:** 2023-04-20

**Authors:** Szu-Yu Chen, Ming-Hsin Lai, Yi-Ling Chu, Dong-Hong Wu, Chih-Wei Tung, Yue-Jie Chen, Chia-Lin Chung

**Affiliations:** 1 Department of Plant Pathology and Microbiology, National Taiwan University, No. 1, Sec. 4, Roosevelt Rd, Taipei City, 106319 Taiwan; 2 Crop Science Division, Taiwan Agricultural Research Institute, No. 189, Zhongzheng Rd., Wufeng Dist, Taichung City, 413008 Taiwan; 3 Department of Agronomy, National Taiwan University, No. 1, Sec. 4, Roosevelt Rd, Taipei City, 106319 Taiwan

**Keywords:** *Fusarium fujikuroi*, Bakanae resistance, Quantitative trait loci (QTLs), *qBK2.1*, *qBK1.8*, Recombinant inbred lines (RILs), Budda, Taikeng 16, Genotyping-by-sequencing (GBS), Molecular markers

## Abstract

**Background:**

Bakanae disease caused by *Fusarium fujikuroi* is an increasing threat to rice production. The infected plants show symptoms such as elongation, slenderness, chlorosis, a large leaf angle, and even death. Bakanae disease is traditionally managed by seed treatment. However, fungicide-resistant *F. fujikuroi* isolates have emerged in several Asian areas, including Taiwan. This study aimed to identify new bakanae resistance quantitative trait loci (QTLs) and provide molecular markers to assist future breeding.

**Results:**

A population of F_2:9_ recombinant inbred lines (RILs) was derived from the cross between an elite *japonica* Taiwanese cultivar ‘Taikeng 16 (TK16)’ and an *indica* variety ‘Budda’. ‘Budda’ was found highly resistant to all 24 representative isolates of the *F. fujikuroi* population in Taiwan. For the RIL population, 6,492 polymorphic single nucleotide polymorphisms (SNPs) spanning the rice genome were obtained by genotyping-by-sequencing (GBS) technique, and the disease severity index (DSI) was evaluated by inoculation with a highly virulent *F. fujikuroi* isolate Ff266. Trait-marker association analysis of 166 RILs identified two QTLs in ‘Budda’. *qBK2.1* (21.97–30.15 Mb) is a novel and first bakanae resistance QTL identified on chromosome 2. *qBK1.8* (5.24–8.66 Mb) partially overlaps with the previously reported *qBK1*.3 (4.65–8.41 Mb) on chromosome 1. The log of odds (LOD) scores of *qBK1.8* and *qBK2.1* were 4.75 and 6.13, accounting for 4.9% and 8.1% of the total phenotypic variation, respectively. 64 RILs carrying both *qBK1.8* and *qBK2.1* showed lower DSI (7%) than the lines carrying only *qBK1.8* (15%), only *qBK2.1* (13%), or none of the two QTLs (21%). For the future application of identified QTLs, 11 KBioscience competitive allele-specific PCR (KASP) markers and 3 insertion-deletion (InDel) markers were developed.

**Conclusions:**

Compared to other important rice diseases, knowledge of bakanae resistance has been insufficient, which limited the development and deployment of resistant cultivars. The discovery of *qBK2.1* has provided a new source of bakanae resistance. The resistant RILs inheriting good plant type, good taste, and high yield characteristics from ‘TK16’ can be used as good resistance donors. Our newly developed markers targeting *qBK2.1* and *qBK1.8* can also serve as an important basis for future fine-mapping and resistance breeding.

**Supplementary Information:**

The online version contains supplementary material available at 10.1186/s40529-023-00375-y.

## Background

Rice is an important staple food for more than half of the people worldwide. It was estimated that farmers can lose about 37% of rice production due to diseases and pests (International Rice Research Institute, Rice Knowledge Bank, http://www.knowledgebank.irri.org). Among the diseases causing considerable reduction in rice quality and yield, Bakanae disease, a seed-borne disease caused by *Fusarium fujikuroi*, is widely distributed in rice-growing areas (Gupta et al. 2015). In recent years, bakanae disease is increasingly important in many Asian countries. In India, it is a severe threat to basmati rice. Disease incidence was as high as 40% in northern India according to a survey from 2006 to 2012 (Gupta et al. 2014), and was 1-25.5% in Odisha state from 2016 to 2018 (Raghu et al. 2018). In Malaysia and Indonesia, disease incidence ranged from 0.5 to 12.5% from 2004 to 2005 (Zainudin et al. 2008). In the affected areas of Bangladesh, the estimated incidence was 19.1%, and the yield loss was 10.85% (Hossain et al. 2011). In Taiwan, the incidence of bakanae disease was also reported to reach 15% in Taitung in 2009 (Chu et al. 2010) and 30.4% in Miaoli in 2012 (Zheng et al. 2016).

*F. fujikuroi*-infected seedlings show several types of symptoms, such as elongation, slenderness, chlorosis, large leaf angle, and even death. It can also cause a reduction of effective tillers, which affects rice yield (Hossain et al. 2011). Rice panicles could be infected during the flowering stage (Ou 1985), so the pathogen can be disseminated through contaminated seeds. To manage bakanae disease, chemical fungicides have long been used for seed treatment. However, *F. fujikuroi* isolates resistant to benzimidazole, prochloraz, tebuconazole, and phenamacril have been reported in Korea, Taiwan, and China (Kim et al. 2010; Chen et al. 2014; Chen et al. 2016; Hou et al. 2018). In China, the frequency of phenamacril-resistant isolates increased from 18% in 2017 to 47% in 2018 (Wu et al. 2019). Because of the emergence of fungicide-resistant isolates, it is crucial to develop alternative control methods.

Planting resistant cultivars is an effective, economical, and environmental-friendly disease control strategy. To breed for resistant cultivars, it is essential to have sufficient information on the genetics of disease resistance. Bakanae-resistant varieties have been identified from large-scale screenings by several research teams (Fiyaz et al. 2014; Kim et al. 2014; Hur et al. 2016; Chen et al. 2019). At least 29 quantitative trait loci (QTLs) controlling bakanae resistance have been identified by linkage mapping and genome-wide association mapping. Thirteen of them are located on chromosome 1 (*qBK1_628091*, *qBK1*^*Z*^, *qBK1.2*, *qBK1.3*, *qBK1*^*WD*^, *qFfR1*, *qBK1*, *qBK1.1*, *qB1*, *qBK1.4*, *qBK1.5*, *qBK1.6*, and *qBK1.7*), and 16 are located on chromosomes 3, 4, 6, 8, 9, 10, and 11 (*qBK3.1*, *qBK3.2*, *qBK4_31750955*, *qBK4.1*, *qBK4*^*T*^, *qFfR6*, *qBK6.1*, *qBK6.2*, *qBK6.3*, *qBK8.1*, *qFfR9*, *qB10*, *qBK10.1*, *qBK10.2*, *qBK10.3*, and *qBK11.1*) (Yang et al. 2006; Hur et al. 2015; Fiyaz et al. 2016; Volante et al. 2017; Ji et al. 2017; Lee et al. 2018, 2019, 2021, 2022; Cheon et al. 2019; Chen et al. 2019; Kang et al. 2019).

This study aimed to identify new resistant QTLs related to bakanae disease and provide molecular markers to assist future breeding. The *indica* rice variety ‘Budda’ is an Indian landrace characterized by drought tolerance and great early vigor (Khera et al. 2012). ‘Budda’ was found to be highly resistant to bakanae, according to the inoculation of 141 and 729 rice accessions with a highly virulent isolate *F. fujikuroi* Ff266 (M.-H. Lai, unpublished data). Chen et al. (2016) analyzed the genetic structure of the *F. fujikuroi* population in Taiwan and evaluated the virulence of 24 representative isolates. Among eight varieties tested, ‘Budda’ exhibited great resistance to all 24 isolates, and ‘Taikeng 16 (TK16)’, a Taiwanese *japonica* cultivar, showed moderate resistance/susceptibility. ‘TK16’ is an elite cultivar with high adaptability, high yield, good taste, brown planthopper resistance, and low pre-harvest sprouting rate (Lee et al. 1998). To find out the genetic regions controlling bakanae resistance in ‘Budda’ and also improve the resistance of ‘TK16’, we generated an F_9_ recombinant inbred line (RIL) population from the cross of ‘TK16’ and ‘Budda’. High-density single nucleotide polymorphisms (SNPs) spanning the rice genome were obtained by the genotyping-by-sequencing (GBS) technique (Elshire et al. 2011), and the disease severity index was evaluated by artificial inoculation and visual rating. After mapping QTLs by linkage analysis, we also developed markers polymorphic between ‘Budda’ and ‘TK16’ and identified potential defense-related genes in the candidate QTL regions. The resistant QTLs, newly developed markers, and resistant lines derived from the mapping population can be used for resistance breeding.

## Methods

### Plant materials

A population of 183 F_9_ RILs was developed from the cross of ‘TK16’ × ‘Budda’ using the single-seed descent method at the experimental farm of Taiwan Agricultural Research Institute. The leaves of F_8_ lines were collected for genotyping, and bakanae resistance was evaluated using F_9_seedlings.

### Evaluation of bakanae disease resistance

*F. fujikuroi* Ff266, a highly virulent isolate originated from a diseased rice plant in Ilan in 2012, was provided by Dr. Chi-Yu Chen at National Chung-Hsing University. Bakanae resistance was evaluated by inoculation with *F. fujikuroi* Ff266 following the methods of Chen et al. (2019). Rice seeds were disinfected in sterile dH_2_O at 60^o^C for 10 min, then soaked in water. *F. fujikuroi* was cultured on 1/2 potato dextrose agar at 25 °C for 4 days. Then, spore suspension was collected using sterile dH_2_O and adjusted to 10^5^ spores/mL. The 4-day-old pre-germinated seeds were then immersed in sterile water (as a control) or the spore suspension and shaken overnight. The seeds were sown in Akadama soil and cultivated in a walk-in chamber (32/28°C day/night temperature, 12/12-h light/dark photoperiod). The inoculation experiment was conducted following a randomized complete block design in three independent trials, each containing 12 seedlings per line. The 12 seedlings were cultivated in a pot (L × W × H = 3.5 × 4.5 × 5.5 cm). At 21 days after inoculation, each *F. fujikuroi*-inoculated seedling was compared with the control and rated on a 0–3 scale (0: symptomless; 1: the presence of one symptom; 2: the presence of two symptoms; 3: seedling death) (Chen et al. 2016). The overall disease severity index (DSI) was calculated as: [(∑scale × No. of seedlings with the scale) / (Maximum scale × Total no. of seedlings)] × 100% (Chen et al. 2019).

### Genotyping-by-sequencing

Rice genomic DNA was extracted from the leaves of the 183 F_8_ RILs and two parents following a cetyltrimethylammonium bromide (CTAB) extraction protocol (Doyle and Doyle 1987). DNA pellet was resuspended with 50 µL Tris-EDTA buffer containing 1 µL of 10 mg/mL RNase A. DNA concentration was quantified using Quant-iT™ PicoGreen™ dsDNA Assay Kit (Invitrogen, MA, USA) and SpectraMax i3x Multi-Mode Microplate Reader (MOLECULAR DEVICES, San Jose, CA, USA) following the manufacturer’s instructions. The genotyping-by-sequencing procedure was conducted following the protocol developed by Elshire et al. (2011) with slight modifications. Briefly, 2 µL of the barcode adaptor was put into each well of the 96-well plate. 100 ng of DNA was added and digested with ApeKI (New England Biolabs, Ipswich, MA, USA) for two hours at 75^o^C. The adaptor was then ligated with the DNA sample by T4 DNA ligase (New England Biolabs, Ipswich, MA, USA) for one hour at 22^o^C and inactivated for 30 min at 65^o^C. Samples in each 96-well plate were pooled and cleaned up using the QIAquick PCR Purification Kit (Qiagen). PCR was performed to increase fragments for sequencing. Subsequently, the PCR product was cleaned up and resuspended with EB buffer. Library quality was checked using Agilent 2100 Bioanalyzer (Agilent Technologies, CA, USA) according to Agilent High Sensitivity DNA Kit Guide. Each 96 multiplexed library was sequenced as 150 bp single-end reads on one lane on the Illumina HiSeq 2500 platform at the NGS High Throughput Genomics Core at Academia Sinica.

### Genetic map construction and linkage mapping

Raw reads generated from GBS were processed according to TASSEL GBS pipeline v3.0 (Glaubitz et al. 2014) and aligned to the rice reference genome IRGSP-1.0 (MSU7 Nipponbare reference genome). Markers with missing data in any line were removed. Lines with high heterozygosity (proportion heterozygous ≥ 0.1) were excluded. The SNP data was transformed to ABH format in TASSEL 5.2.60 (Bradbury et al. 2007). Crossover points and bin markers were calculated using SNPbinner (Gonda et al. 2019).

Genetic map construction and linkage mapping were conducted using R/qtl (Broman et al. 2003). The construction of the genetic map was based on the Kosambi map function. The QTLs were identified by interval mapping with the threshold determined based on 1,000 permutations at 5% and 1% significance levels. The interval of each QTL was defined using the Bayesian credible interval at 0.95 probability coverage. Epistatic interactions were calculated using R/qtl (effectplot function), with each QTL represented by the bin marker nearest the QTL peak. To investigate the possible causal genes involved in bakanae resistance, gene function descriptions were acquired from Rice Annotation Project Database (RAP-DB).

### Marker development

For the future application of identified QTLs, KBioscience competitive allele-specific PCR (KASP) and insertion-deletion (InDel) markers across the candidate genetic regions were designed (Supplemental Tables 1 and Supplemental Table 2). KASP markers were designed following the methods of Jatayev et al. (2017) and Wang et al. (2019) with modifications. SNPs were acquired from the GBS result. Briefly, allele-specific primers and common reverse primers were designed using WASP Web-based Allele Specific Primer design tool (http://bioinfo.biotec.or.th/WASP) or BatchPrimer3 (https://wheat.pw.usda.gov/demos/BatchPrimer3/) (Wangkumhang et al. 2007; You et al. 2008). Then, a mismatched nucleotide was substituted at the fourth position from the 3’ end. The sequences (GAAGGTGACCAAGTTCATGCT and GAAGGTCGGAGTCAACGGATT) corresponding to FAM or VIC/HEX probe were added to the 5’ end of two forward primers. The primers were diluted to 15 µM and mixed with the ratio listed in Supplemental Table 1. Each 10 µL reaction contained 5 µL KAPA PROBE FAST qPCR Master Mix (2X) Universal (KAPA Biosystems, MA, USA), 0.14 µL KASP primer mix, 2 µL of DNA (15 ng/µL), 0.3 µL of 4 µM fluorescent probe, and 2.26 µL ddH_2_O. PCR reaction was performed on CFX Connect Real-Time PCR Detection System (BioRad, CA, USA). The thermal cycling parameters were 95 °C for 3 min followed by 10 cycles of 95 °C for 20 s, 61 °C for 20 s (-0.6 °C/cycle), and 72 °C for 1 s; then 40 cycles of 95 °C for 20 s, and 55 °C for 40 s.

The InDel markers (Supplemental Table 2) were developed based on the sequence difference between *indica* and *japonica* rice cultivars. The whole genome sequences of the *indica* rice IR64 and the *japonica* rice Nipponbare were used. Genome alignment was conducted in the Rice SNP-Seek Database (https://snp-seek.irri.org/) and primers were designed using Primer3Plus (Untergasser et al. 2007). Each PCR reaction was performed in a total volume of 10 µL, containing 1 µL of genomic DNA (20 ng/µL), 5 µL of 2X Master Mix Red PCR buffer (Ampliqon, Denmark), 0.1 µL of 10 µM forward primer, 0.1 µL of 10 µM reverse primer, and 3.8 µL of ddH_2_O. The thermal cycling parameters were 95 °C for 5 min followed by 32 cycles of 95 °C for 30 s, 61 °C for 30 s, and 72 °C for 1 min, and a final extension step at 72 °C for 5 min. PCR products were electrophoresed in a 2% agarose gel in 0.5X Tris-borate-EDTA buffer for 20 min at 100 V. Gels were stained with ethidium bromide (5 × 10^− 4^ mg/mL) for 10 min, destained with ddH_2_O for 10 min, then photographed using the Gel Documentation-VideGel VGIS-4 system (Top Bio Co.).

## Results

### Evaluation of bakanae resistance in the RIL population

**Fig. 1:**
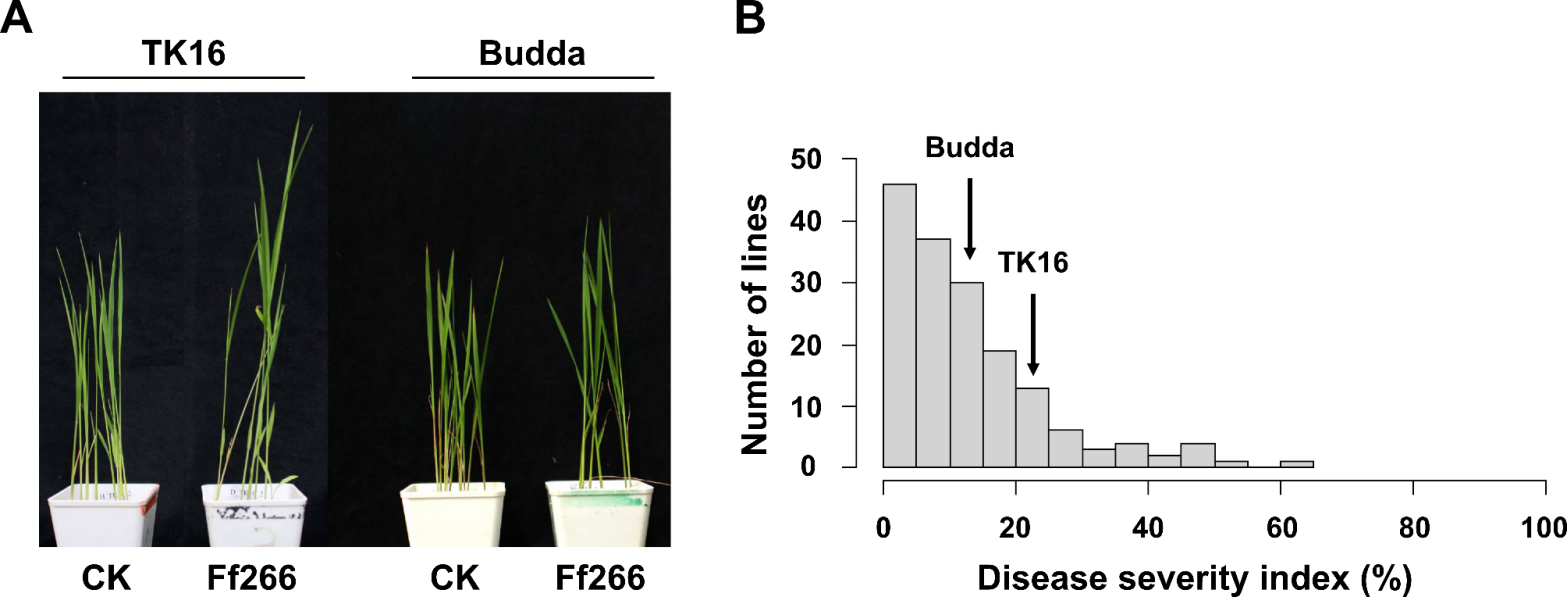
Bakanae resistance of ‘TK16’, ‘Budda’, and the derived recombinant inbred lines (RILs). **A**, Symptoms of ‘TK16’ and ‘Budda’ inoculated with *Fusarium fujikuroi* Ff266 at 21 days post-inoculation. **B**, Frequency distribution for disease severity index in the RIL population derived from ‘TK16’ × ‘Budda’. The arrows indicate the means of disease severity index (DSI) of ‘TK16’ and ‘Budda’

To identify the bakanae resistance in ‘Budda’, the RIL population derived from ‘TK16’ × ‘Budda’ was artificially inoculated with *F. fujikuroi* Ff266. Major types of symptoms observed from the inoculated parental lines and the RIL population were elongation and slenderness. The means of DSI of ‘Budda’ and ‘TK16’ were 14% and 21.4%, respectively. The distribution of DSI for 183 RILs tested ranged from 0 to 63.8% (Fig. 1), with most lines exhibiting moderate resistance to bakanae disease. The RIL population showed transgressive segregation, with some lines displaying greater resistance or susceptibility than the parental lines.

### Genetic map construction

**Fig. 2:**
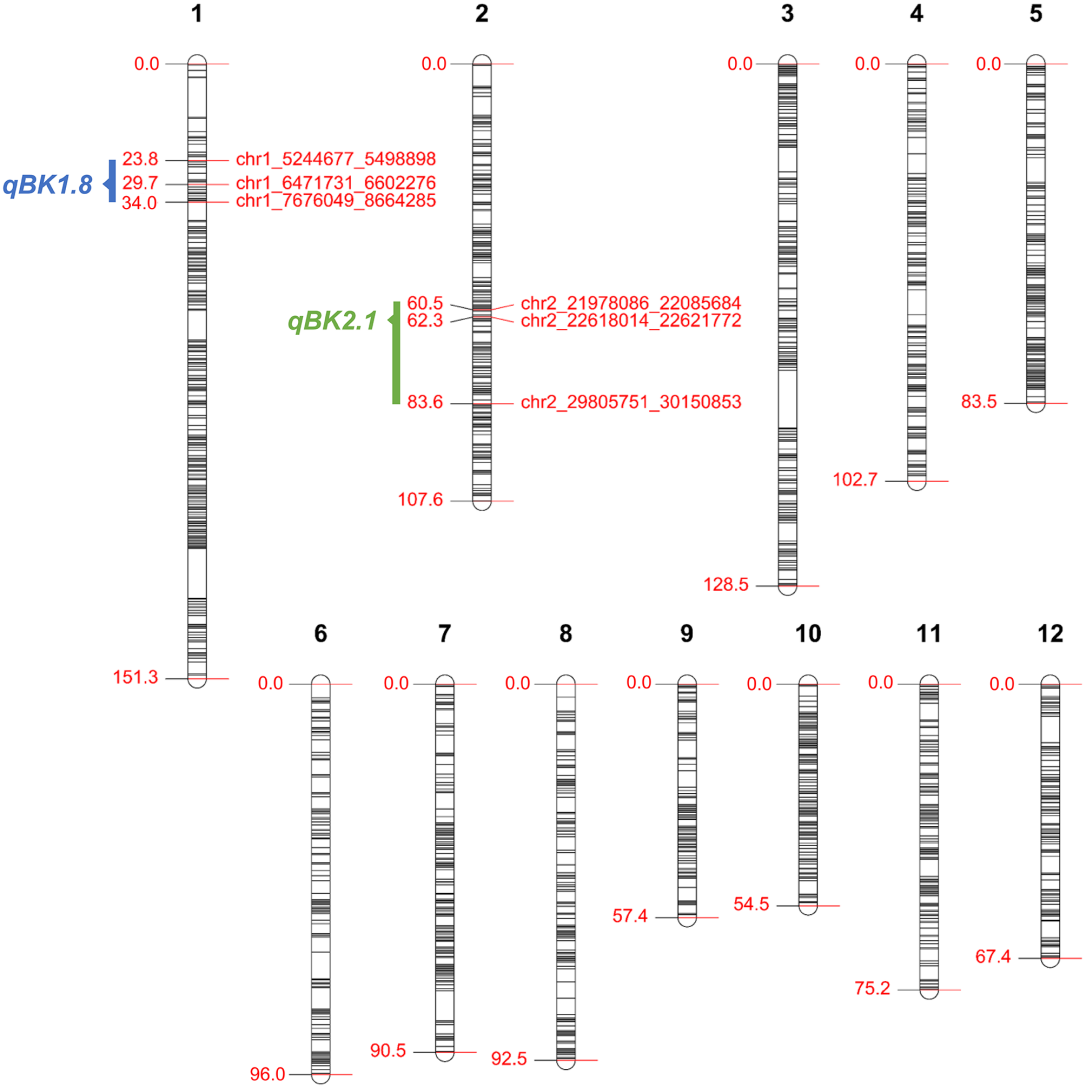
Genetic map of 1,622 bin markers. The numbers on the left of chromosomes are genetic map positions. The candidate intervals of *qBK1.8* and *qBK2.1* are labeled as blue and green bars, respectively. Border markers and the markers nearest the QTL peaks are labeled on the right of the chromosomes

A total of 184,170 SNPs with polymorphism between ‘TK16’ and ‘Budda’ were generated from the GBS result. After the exclusion of 17 lines with high heterozygosity (proportion heterozygous ≥ 0.1), a total of 166 lines were used for subsequent analysis. Following the removal of SNPs with missing data for any lines, 6,492 SNPs were retained to construct the linkage map. With the distortion value set at 0.001 and the minimum size of each bin calculated based on 1,000 bp, a total of 1,622 bin markers defined by SNPbinner were used for genetic map construction by R/qtl. The genetic map spanned 1107.1 cM in total with an average density of ~0.68 cM per bin marker. Segregation distortions were identified in chromosomes 3 (10.5–24.7 Mb), 6 (3.7–6.2 Mb), and 7 (3.3–22.0 Mb) (Fig. 2; Table 1).

**Table 1:** Number of polymorphic SNPs on different chromosomes

Chromosome	No. of SNPs	No. of bins	Genetic length (cM)	Average distance (cM)
1	868	225	151.3	0.67
2	664	173	107.6	0.62
3	695	182	128.5	0.71
4	592	139	102.7	0.74
5	523	133	83.5	0.63
6	520	110	96.0	0.87
7	526	129	90.5	0.70
8	475	119	92.5	0.78
9	383	85	57.4	0.67
10	483	108	54.5	0.50
11	449	119	75.2	0.63
12	314	100	67.4	0.67
**Total**	6,492	1,622	1107.1	0.68

### Detection of QTLs for bakanae resistance

**Fig. 3:**
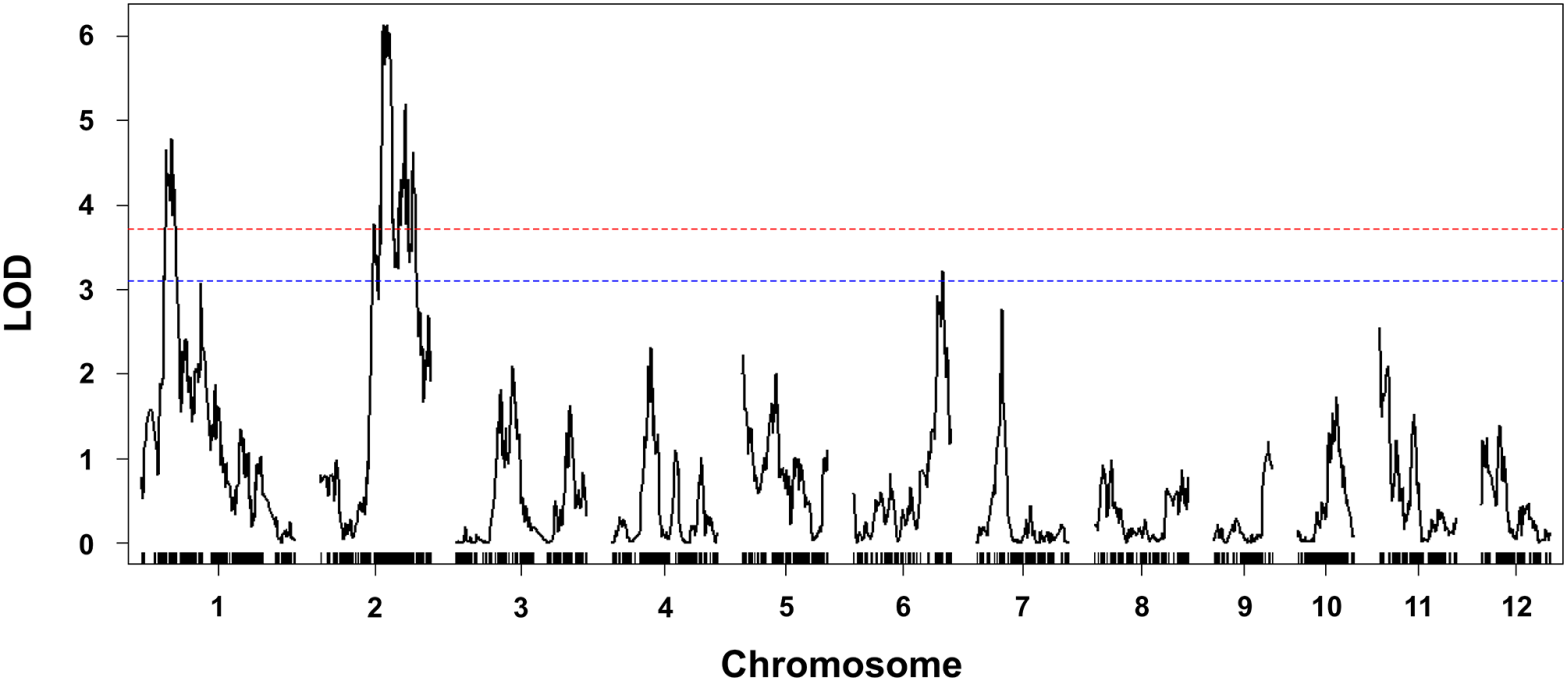
Linkage mapping in the population of 166 recombinant inbred lines (RILs) derived from ‘TK16’ × ‘Budda’. The horizontal line represents the logarithm of odds (LOD) thresholds at 95% (blue) and 99% (red) confidence level based on 1000 permutations

**Fig. 4:**
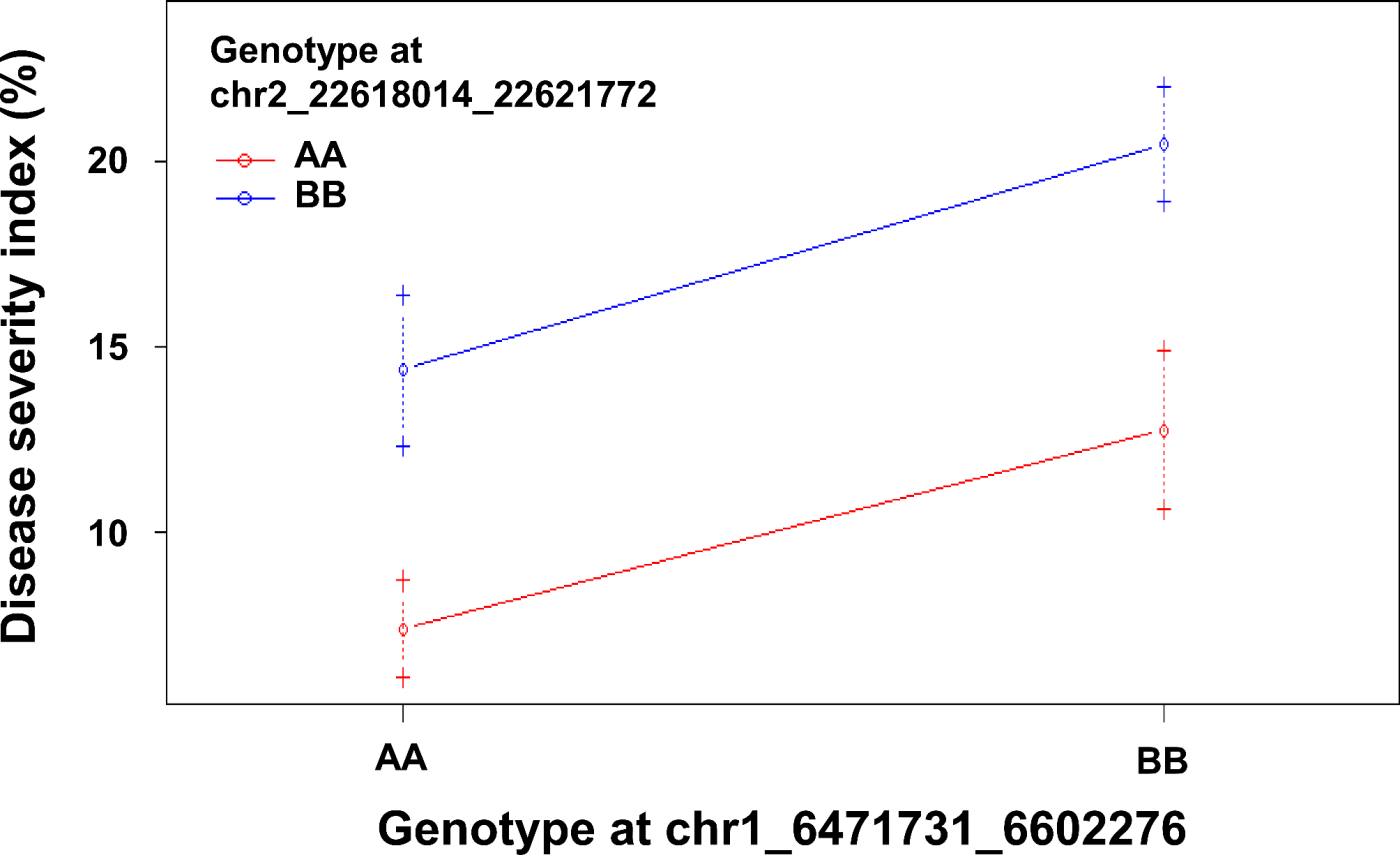
Interaction plot for *qBK1.8* and *qBK2.1.* chr1_6471731_6602276 and chr2_22618014_22621772 are the bin markers nearest the peaks of *qBK1.8* and *qBK2.1*, respectively. Genotypes AA and BB are ‘Budda’ and ‘TK16’ homozygotes, respectively

QTLs associated with bakanae resistance were identified using interval mapping. The LOD thresholds at 95% and 99% confidence levels were 3.10 and 3.72, respectively. Two QTL peaks (LOD > 3.72) were detected on chromosomes 1 and 2 (Table 2; Fig. 3). The resistance effect of both QTLs was from ‘Budda’ alleles. The most significant QTL (LOD = 6.13), *qBK2.1*, is a novel QTL located between 21,978,086 to 30,150,853 bp on chromosome 2. The bin marker nearest the *qBK2.1* peak (62.3 cM) was at 22,618,014 to 22,621,772 bp. The other minor QTL, *qBK1.8*, was identified between 5,244,677 to 8,664,285 bp on chromosome 1. *qBK1.8* is co-localized with a previously reported *qBK1.3* (Fiyaz et al. 2016). The bin marker nearest the *qBK1.8* peak (29.7 cM) was at 6,471,731 to 6,602,276 bp. *qBK2.1* and *qBK1.8* explained 8.1% and 4.9% of phenotypic variation, respectively. No significant epistatic interaction was observed between *qBK2.1* and *qBK1.8* (Fig. 4). Nonetheless, 64 RILs carrying both *qBK1.8* and *qBK2.1* showed lower disease incidence (15.4%) and DSI (7%) than those carrying either *qBK1.8* (disease incidence = 29.2%, DSI = 15%) or *qBK2.1* (disease incidence = 25.2%, DSI = 13%); 46 RILs with neither *qBK1.8* nor *qBK2.1* showed higher disease incidence (40.4%) and DSI (21%).

**Table 2:** Significant QTLs identified from 166 recombinant inbred lines (RILs) of ‘TK16’ × ‘Budda’

QTL	Chromosome	LOD score	QTL region (cM)	QTL region (bp)^a^	Interval size (bp)	Bin marker nearest the QTL peak ^b^	Variation explained (%)	Additive effect (%)^b^	Number of genes in the QTL
*qBK2.1*	2	6.13	60.51–83.59	21,978,086 − 30,150,853	8,172,767	chr2_22618014_22621772	8.1	3.7	1,060
*qBK1.8*	1	4.75	23.82–33.98	5,244,677-8,664,285	3,419,608	chr1_6471731_6602276	4.9	2.9	442

^a^ The genomic position is based on the IRGSP-1.0 reference genome

^b^ The peaks of *qBK2.1* and *qBK1.8* were identified at 62.3 and 29.7 cM, respectively. The bin markers nearest the QTL peaks were located at 22,618,014–22,621,772 bp on chromosome 2 and 6,471,731-6,602,276 bp on chromosome 1

^c^ Positive value indicates the resistant effect of the ‘Budda’ allele

### Candidate genes in QTL regions

A total of 1,060 and 442 genes are located within *qBK2.1* and *qBK1.8*, respectively. Among these genes, 12 genes contain a leucine-rich repeat (LRR) domain (*Os01g0223600*, *Os01g0239700*, *Os02g0593600*, *Os02g0597300*, *Os02g0609900*, *Os02g0610000*, *Os02g0614966*, *Os02g0615400*, *Os02g0636400*, *Os02g0647300*, *Os02g0658500*, and *Os02g0695050*), one gene contains an NB-ARC domain (*Os02g0612200*), and four genes are involved in disease resistance (*Os01g0229400*, *Os02g0629400*, *Os02g0632800*, and *Os02g0700700*). *Os02g0629400* and *Os02g0632800* were annotated to regulate the resistance to bacterial leaf streak and rice blast, respectively. Moreover, candidate genes related to phytohormones were also found. Three genes (*Os01g0209700*, *Os02g0580300*, and *Os02g0643200*) are associated with gibberellin signaling or catabolism. Four genes (*Os02g0626100*, *Os02g0626400*, *Os02g0626600*, and *Os02g0627100*) and two genes (*Os01g0246700* and *Os02g0629800*) are involved in salicylic acid (SA) biosynthesis and jasmonic acid (JA) signaling, respectively. Seven genes (*Os01g0224700*, *Os01g0231000*, *Os01g0236300*, *Os02g0610950*, *Os02g0628600*, *Os02g0643800*, and *Os2g0723400*) are related to auxin regulation.

### Marker development

Six KASP markers were developed for *qBK1.8*, and 5 KASP and 3 InDel markers were developed for *qBK2.1* (Supplemental Tables 1 and Supplemental Table 2). The amplification of KASP markers showed that the genotypes of ‘Budda’ (FAM-signal), ‘TK16’ (HEX/VIC-signal), and heterozygotes could be clearly differentiated into three groups (Supplemental Fig. 1 and Supplemental Fig. 2). The three newly developed InDel markers amplified specific ‘Budda’ and ‘TK16’ fragments of expected sizes (Supplemental Fig. 3). The product sizes of Indel_chr2_1 were 1,051 bp for Budda and 399 bp for TK16; Indel_chr2_3 were 384 bp for Budda and 751 bp for TK16; Indel_chr2_5 were 366 bp for Budda and 608 bp for TK16.

## Discussion

The infection of *F. fujikuroi* usually causes complex morphological changes and a more significant variation of disease reactions among individual plants. Derived from consecutive inbreeding, the RIL population has high levels of recombination and homozygosity and can be used for repeated phenotyping, which largely increases the power of QTL detection (Burr and Burr 1991; Broman 2005). Four bakanae resistance QTLs, i.e., *qBK1.1*, *qBK1.2*, *qBK1.3*, and *qBK3.1*, were mapped using a population of F_14_ RILs (Fiyaz et al. 2016). *qBK1*^*WD*^ and *qBK1*^*Z*^ on chromosome 1 were fine-mapped by using F_4:5_ and F_2:9_ RILs, respectively. (Lee et al. 2018, 2021). Recently, *qBK4*^*T*^ was mapped by using BC_1_F_8_ RILs (Lee et al. 2022). In this study, to uncover the genetics underlying the high bakanae resistance in ‘Budda’, we generated a population of ‘TK16’ × ‘Budda’ F_9_ RILs. In combination with high-density SNP genotypes generated by GBS and more precise disease evaluation in a controlled environment, we have successfully identified two QTLs with relatively minor effects (8.1% and 4.9% of variation explained). *qBK2.1* is a novel resistant QTL and the first bakanae resistance QTL identified on chromosome 2; *qBK1.8* (5.24–8.66 Mb) is co-localized with the previously reported *qBK1.3* (4.65–8.41 Mb) on chromosome 1 (Fiyaz et al. 2016). In the future, narrowing down the candidate regions through fine-mapping will precisely identify resistance gene(s) in ‘Budda’ to facilitate resistance breeding.

Plants have evolved different mechanisms to perceive and respond to pathogen infection. The defense-related candidate genes located in the regions of *qBK2.1* and *qBK1.8* are worthy of further investigation. The 12 LRR-containing proteins may function as pattern recognition receptors (PRRs) on the cell surface or nucleotide-binding domain leucine-rich repeat (NLR) proteins intracellularly to recognize pathogen effectors and induce defense responses (Yuan et al. 2021). *Os02g0632800* encodes a wall-associated kinase OsWAK14, which was associated with quantitative resistance to rice blast by loss-of-function mutants (Delteil et al. 2016). The identified JA-, GAs-, and auxin-related genes might affect bakanae resistance and symptom development. The exogenous application of methyl jasmonate (MeJA) could delay the symptom development in a susceptible cultivar (Cheng et al. 2020). Auxin signaling has been implicated in regulating leaf angle (Zhang et al. 2015), and GAs control plant height and internode elongation (Takahashi et al. 1991; Ayano et al. 2014). The interplay of phytohormones and their effects on bakanae resistance remains to be clarified.

Three segregation distortion regions on chromosomes 3, 6, and 7 were observed for the ‘TK16’ × ‘Budda’ RIL population. The phenomenon could be attributed to the reproductive barriers of *indica-japonica* hybrids. Segregation distortion on chromosome 3 might be associated with impaired male functions such as pollen sterility and spikelet sterility (Reflinur et al. 2014). Female factors such as embryo sac fertility, embryo sac abortion, and female gametogenesis might be responsible for the segregation distortion on chromosome 6 (Reflinur et al. 2014). Besides, a locus controlling temperature-sensitive genic male sterility was reported on chromosome 7 (Yamaguchi et al. 1997). Segregation distortion would increase the difficulty of detecting QTLs in these regions. To discover potential QTLs in the segregation distortion region, a new mapping population should be developed by crossing between ‘Budda’ and other *Indica* rice varieties.

Pyramiding of resistant QTLs could strengthen rice to defend against bakanae disease. Lee et al. (2018) observed that the mean proportions of healthy plants for the F_4_ RILs with both *qBK*^*WD*^ and *qBK1* (80.2%) was higher than that with only *qBK*^*WD*^ (66.1%) or *qBK1* (55.5%) and that without resistance QTLs (35.3%). Similarly, in our RIL population, the lines harboring *qBK2.1* and *qBK1.8* showed the highest level of resistance and the lowest disease incidence. We are currently working to evaluate the agronomic traits of the RILs containing resistant alleles at *qBK2.1* and *qBK1.8*. The resistant lines inheriting good plant type, good taste, and high yield characteristics from ‘TK16’ can potentially be released as new cultivars or used as resistance donors.

## Conclusions

Bakanae disease is an important and emerging epidemic of rice worldwide. The occurrence of fungicide resistance made the conventional seed treatment ineffective. However, compared to other important rice diseases such as blast and bacterial blight, knowledge of bakanae resistance has been insufficient, which limited the development and deployment of resistant cultivars. In this study, to more accurately map QTLs controlling the resistance of ‘Budda’, we constructed a ‘TK16’ × ‘Budda’ F_9_ RIL population and used the GBS technique to generate genome-wide high-density SNPs. We also adopted strict criteria for linkage analysis, including the removal of lines with high heterozygosity and the SNPs with missing data for any lines. The discovery of *qBK2.1* and *qBK1.8*, particularly the novel *qBK2.1*, has provided a new source of bakanae resistance. Our newly developed KASP and InDel markers targeting *qBK2.1* and *qBK1.8* are ready for use in future fine-mapping and resistance breeding.

## Electronic supplementary material

Below is the link to the electronic supplementary material.

Supplementary Material 1

## Data Availability

The datasets used and/or analysed during the current study are available from the corresponding author on reasonable request.

## Abbreviations

CTAB: cetyltrimethylammonium bromide
DSI: disease severity index
GBS: genotyping-by-sequencing
InDel: insertion-deletion
KASP: KBioscience competitive allele-specific PCR
LOD: log of odds
LRR: leucine-rich repeat
NLR: nucleotide-binding domain leucine-rich repeat
PRR: pattern recognition receptor
QTL: quantitative trait locus
RIL: recombinant inbred line
SNP: single nucleotide polymorphism
TK16: Taikeng 16
