# Supplementary material for: Identification of qBK2.1, a novel QTL controlling rice resistance against Fusarium fujikuroi
Source: Bot Stud. 2023 Apr 20;64:11. doi: 10.1186/s40529-023-00375-y (PMC10119339; doi:10.1186/s40529-023-00375-y)
Supplement: Supplementary file 1 — Supplementary Material 1 [file 40529_2023_375_MOESM1_ESM.docx]

**Supplementary materials**

**Supplemental Table S1.** List of KBioscience competitive allele-specific PCR (KASP) markers developed in this study

| **ID** | **Chr.** | **Position**  **(bp)^a^** | **Primer ratio** | | | **Forward primer (F1)** | **Forward primer (F2)** | **Common reverse primer (R)** |
| --- | --- | --- | --- | --- | --- | --- | --- | --- |
|  |  |  | **F1** | **F2** | **R** |  |  |  |
| S1_5230535 | 1 | 5,230,535 | 1.5 | 0.5 | 5.2 | FAM: GAAGGTGACCAAGTTCATGCT  GCAGCACATTGATTTAGCGGT | VIC: GAAGGTCGGAGTCAACGGATT  GCAGCACATTGATTTAGCGGA | TAGAAATTGGTCTCAGCTCCC |
| S1_5244677 | 1 | 5,244,677 | 1 | 1 | 5.2 | FAM: GAAGGTGACCAAGTTCATGCT  CTCAGTGGTCAAAGTCCAATCG | VIC:GAAGGTCGGAGTCAACGGATT  TCTCAGTGGTCAAAGTCCAATCA | GTACATGGAGGCATTGGTCC |
| S1_6140049 | 1 | 6,140,049 | 1.3 | 0.7 | 5.2 | FAM: GAAGGTGACCAAGTTCATGCT  CTCACAGCCTCCACAGCTCA | HEX: GAAGGTCGGAGTCAACGGATT  CACAGCCTCCACAGCTCC | TCCACCACAATTTCACAGCC |
| S1_7301159 | 1 | 7,301,159 | 1 | 1 | 5.2 | FAM: GAAGGTGACCAAGTTCATGCT  AGTGACACGACGATCTCCAC | VIC: GAAGGTCGGAGTCAACGGATT  AGTGACACGACGATCTCCAG | TAGCTGCACGAATGAATAAGCTTC |
| S1_8916384 | 1 | 8,916,384 | 1 | 1 | 5.2 | FAM: GAAGGTGACCAAGTTCATGCT  CATGGTACTACACATCCTCTTTCCA | HEX: GAAGGTCGGAGTCAACGGATT  TGGTACTACACATCCTCTTTCCG | GTTGTCCTTGTATAATTGTACTGCC |
| S1_8979023 | 1 | 8,979,023 | 1 | 1 | 5.2 | FAM: GAAGGTGACCAAGTTCATGCT  CAAACCGAACAACAAGCTCAAA | VIC: GAAGGTCGGAGTCAACGGATT  CAAACCGAACAACAAGCTCAAG | TAACGGACAGGCTGAATCTGTA |
| S2_21851681 | 2 | 21,851,681 | 2 | 0.4 | 5.2 | FAM: GAAGGTGACCAAGTTCATGCT  ACACGCAAGATAGCCAGTCC | VIC: GAAGGTCGGAGTCAACGGATT  ACACGCAAGATAGCCAGTCG | AAATAGTGGCATCATCAGCGA |
| S2_24014506 | 2 | 24,014,506 | 1.5 | 0.5 | 5.2 | FAM: GAAGGTGACCAAGTTCATGCT  AGCGAGTAGTGACACAACCAC | VIC: GAAGGTCGGAGTCAACGGATT  CAGCGAGTAGTGACACAACCAT | CACAAGCAAAGTCCGTCGTC |
| S2_26530187 | 2 | 26,530,187 | 1.5 | 0.5 | 5.2 | FAM: GAAGGTGACCAAGTTCATGCT  ATGGGTCAGTAGATCACAAACG | VIC: GAAGGTCGGAGTCAACGGATT  CATGGGTCAGTAGATCACAAACA | TGCAAGGTCAGCTCATCAGC |
| S2_29133772 | 2 | 29,133,772 | 1.3 | 0.7 | 5.2 | FAM: GAAGGTGACCAAGTTCATGCT  GATTGAACTGACGGCACGGT | HEX: GAAGGTCGGAGTCAACGGATT  ATTGAACTGACGGCACGGC | AGCTAACCCAGCACAGGTGT |
| S2_30299957 | 2 | 30,299,957 | 1.3 | 0.7 | 5.2 | FAM: GAAGGTGACCAAGTTCATGCT  GTGAGTTTTTCAGCAACAAGGACT | HEX: GAAGGTCGGAGTCAACGGATT  TGAGTTTTTCAGCAACAAGGACC | TGGTTGCTTCAATGTCTGATGT |

^a^ The genomic position is based on the IRGSP-1.0 reference genome.

**Supplemental Table S2.** List of insertion-deletion (InDel) markers developed in this study

| **ID** | **Chr.** | **Position**  **(bp)^a^** | **Forward primer (5’→3’)** | **Reverse primer (5’→3’)** | **Product size (bp)** | |
| --- | --- | --- | --- | --- | --- | --- |
|  |  |  |  |  | **Budda** | **TK16** |
| Indel_chr2_1 | 2 | 22,337,524 | ACGTATGCTTCAAGTGCCCA | CAGAAGGCACCCCTATGCTT | 1051 | 399 |
| Indel_chr2_3 | 2 | 28,092,599 | CAGTGGAATCGTTGGTGATG | ACCACGTCGTAATCCGGTAA | 384 | 751 |
| Indel_chr2_5 | 2 | 30,059,190 | TCAATCTTGGCAAGCCCTAC | GTCCTAGACGACGACCATGC | 366 | 608 |

^a^ Position indicates the first nucleotide position of forward primer based on the IRGSP-1.0 reference genome.


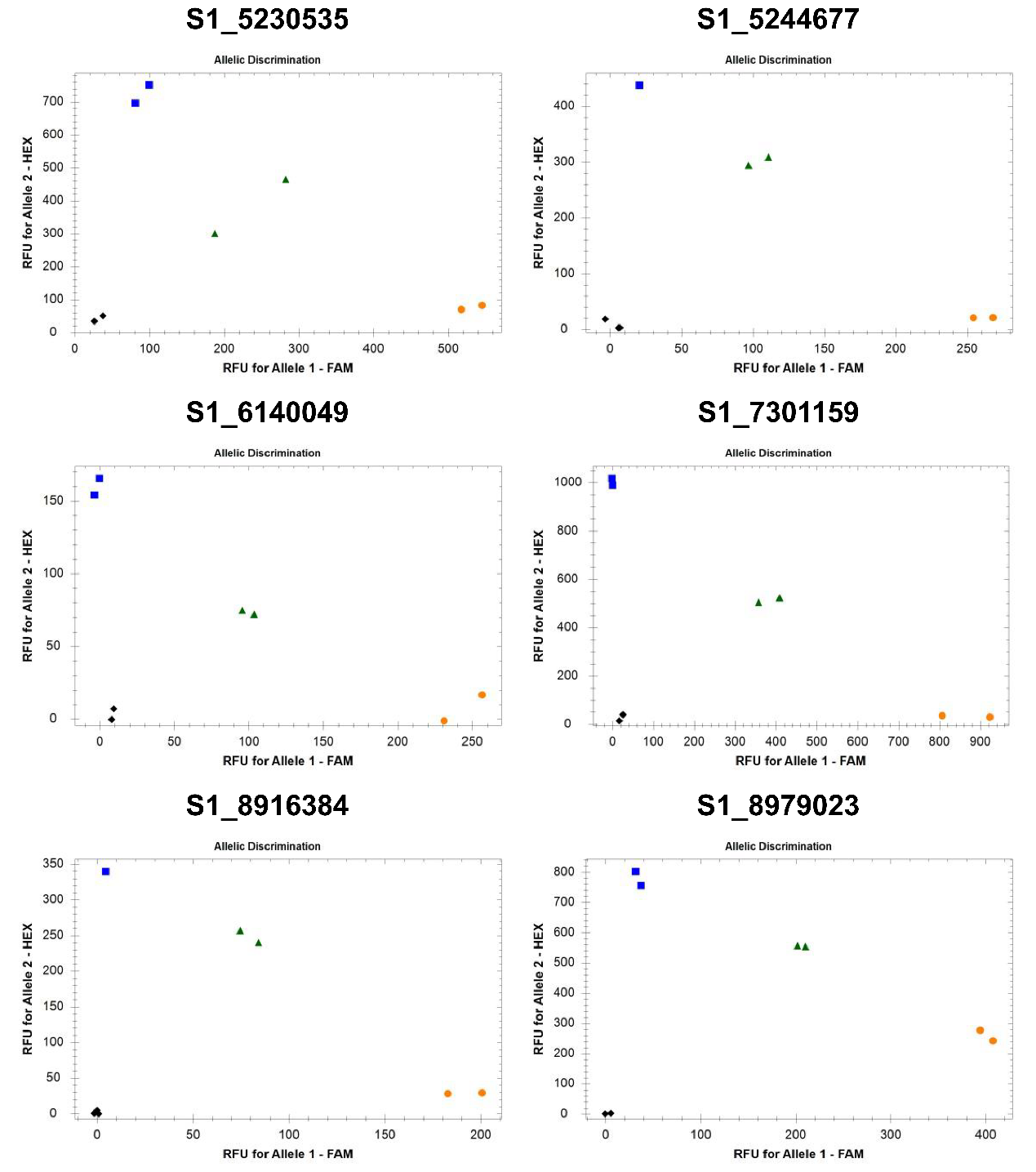


**Supplemental Fig. S1.** Polymorphic KBioscience competitive allele-specific PCR (KASP) markers developed for detecting *qBK1.8*. Square: HEX/VIC-signal, representing the ‘TK16’ genotype; triangle: heterozygous genotype; circle: FAM-signal, representing the ‘Budda’ genotype; diamond: No template control (NTC).


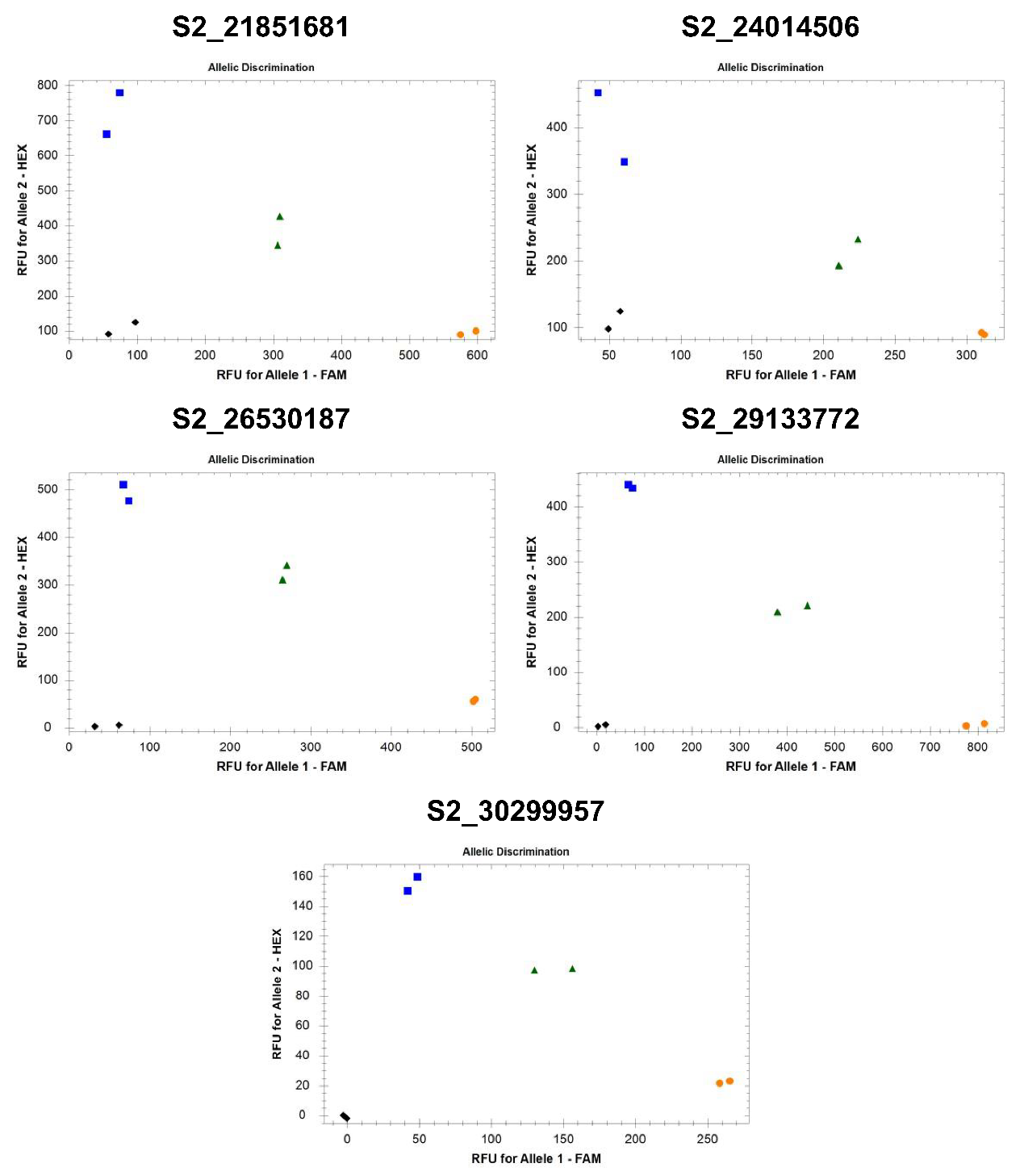


**Supplemental Fig. S2.** Polymorphic Kbioscience competitive allele-specific PCR (KASP) markers developed for detecting *qBK2.1*. Square: HEX/VIC-signal, representing the ‘TK16’ genotype; triangle: heterozygous genotype; circle: FAM-signal, representing the ‘Budda’ genotype; diamond: No template control (NTC).


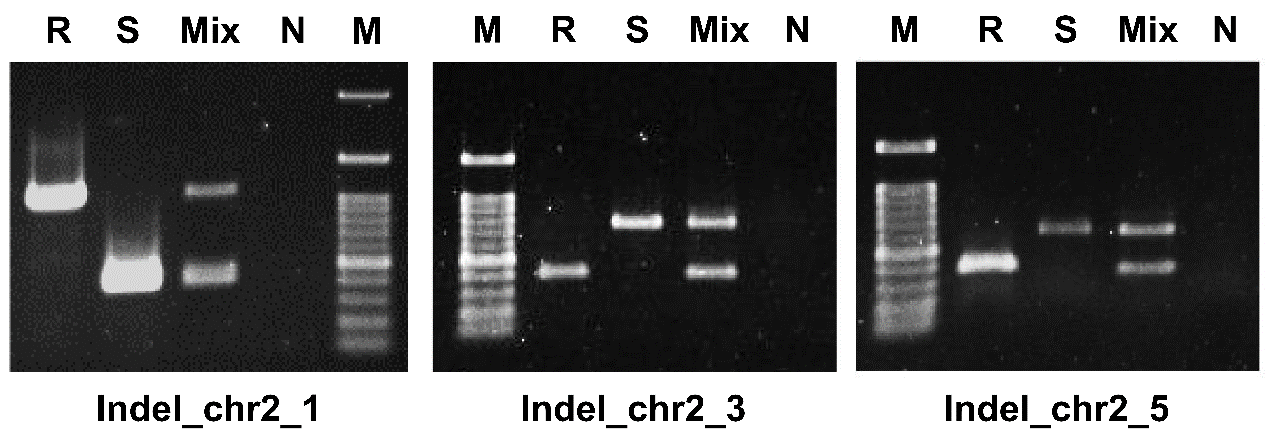


**Supplemental Fig. 3.** Insertion-deletion (InDel) markers developed for detecting *qBK2.1*. R, resistant cultivar ‘Budda’; S, susceptible cultivar ‘Taikeng 16 (TK16)’; Mix, equally mixed DNA of ‘Budda’ and ‘TK16’; N, negative control; M, Omics 100bp Plus DNA RTU Ladder.
